# Energy Availability and Interstitial Fluid Glucose Changes in Elite Male Japanese Triathletes during Training Camp: A Case Study

**DOI:** 10.3390/nu16132048

**Published:** 2024-06-27

**Authors:** Chiyori Hiromatsu, Kazushige Goto

**Affiliations:** Graduate School of Sports and Health Science, Ritsumeikan University, Shiga 525-8577, Japan; sh0041ve@ed.ritsumei.ac.jp

**Keywords:** continuous glucose monitors (CGM), glucose, energy availability (EA), triathletes

## Abstract

This study explored the impact of varying energy availability (EA) on the 24-h interstitial fluid glucose concentration (IGC) in five elite male Japanese triathletes at a training camp. Measurements of IGC, energy and macronutrient intake, and exercise energy expenditure (EEE) through metabolic equivalents (METs) from training logs were conducted. Three subjects were evaluated over two 4-day periods, and two subjects over one 4-day period. Findings revealed significant correlations of daily mean nocturnal IGC with daily EA (r = 0.553, *p* = 0.001) and energy intake (EI) (r = 0.595, *p* < 0.001). However, no significant correlation was found between mean daily nocturnal IGC and EEE (r = −0.278, *p* = 0.124). Daytime IGC was ≥110 mg/dL for >50% of the time in all subjects, except on 1 day in one subject, and never fell <70 mg/dL. Therefore, daily EA may influence nocturnal IGC in elite male triathletes, although high daytime IGC levels were maintained without hypoglycemia.

## 1. Introduction

Triathletes face high energy demands from daily extended training sessions, including swimming, cycling, and running [1]. Recently, some special nutritional strategies for endurance athletes have been discussed. These strategies adjust the ratio of carbohydrate (CHO) and fat intake, which is the primary source of energy for endurance athletes (e.g., ketogenic diet, “Train-High Sleep-Low”) [2,3]. Rigorous training regimes triathletes experience can lead to reduced energy availability (EA) compared to other athletes [4], defined as dietary energy intake (EI) minus exercise energy expenditure (EEE). This remaining energy supports bodily functions and physiological processes [5]. Persistently low EA (LEA) may harm health outcomes and performance [6], and male endurance athletes in a negative energy balance are at a higher risk of lower fasting blood glucose [7]. Similarly, sedentary women with LEA (<30 kcal/kg FFM/day) experience significant drops in nocturnal and average 24-h blood glucose levels compared to those with adequate EA (45 kcal/kg FFM/day) [8]. Elite endurance athletes also tend to spend more time outside the normoglycemic range, experiencing more frequent hypoglycemia and hyperglycemia compared to controls [9].

Continuous glucose monitors (CGMs) are medical devices that track the dynamics of interstitial fluid glucose concentrations (IGCs) across a 24-h period. IGC has a strong correlation with blood glucose levels [10], making CGMs useful for athletes to monitor glucose fluctuations during their daily routines and identify periods of hypoglycemia or hyperglycemia. This is particularly relevant for endurance athletes, such as triathletes, marathon runners, and race walkers, where monitoring IGC can help detect LEA during training. Nocturnal IGC, less affected by meals and physical activity than daytime IGC, can offer insights into an athlete’s glucose metabolism [11]. Understanding EA is crucial during intensive training camps, where the risk of LEA increases with higher EEE. However, most studies on this topic have focused on non-elite male athletes or were conducted in laboratory settings [12,13,14], and few have explored the relationship between EA and 24-h glucose changes in elite endurance athletes during training camps.

The present study sought to investigate how different levels of EA affect 24-h IGC changes in elite male Japanese triathletes during a training camp. We hypothesized that nocturnal IGC would decrease on days with LEA compared to days with optimal EA. Additionally, we anticipated that hypoglycemia would occur during sleep on days of extremely LEA, but not during daytime, regardless of EA levels.

## 2. Methods

### 2.1. Subjects

Five elite male triathletes [mean ± standard deviation (SD) age: 25.0 ± 2.6 years, height: 175.1 ± 5.9 cm, body weight (BW): 62.9 ± 3.2 kg, % body fat: 9.7 ± 0.5%, body mass index: 21.0 ± 0.4 kg/m^2^] participated in the present study. All participants had international competition experience and were either members or candidates for the national team of Japan. Each athlete was fully informed about the study’s purpose, procedures, and potential risks, and provided written informed consent. The study was approved by the Ethical Committee for Human Experiments at Ritsumeikan University (BKC-LSMH-2023-002 and date of approval 05/2023), in accordance with the Declaration of Helsinki.

### 2.2. Experimental Overview

During the training camp, three subjects (subjects 1–3) were continuously monitored for IGC changes across 4 consecutive days during two separate periods (term A: days A1–A4; term B: days B1–B4; total of 8 days) over 10 months. The remaining two subjects (subject 4 during term A and subject 5 during term B) participated in one period each, with their IGC changes monitored over 4 consecutive days. In total, data from 32 days were collected from all five subjects. Term A spanned 4 consecutive days during a 3-week training camp in April at the beginning of the season. Term B also covered 4 consecutive days but took place during a February training camp, just before the season started, with a schedule that included a higher frequency of prolonged road cycling training days.

During term A, set meals were provided three times daily, with only the staple food portions (e.g., rice, noodles) being adjustable by the subjects. Beyond these three meals, subjects could freely consume other food and beverages. Training sessions began between 8:30 and 9:00 a.m., prompting subjects to have breakfast before training each day. In contrast, during term B, meals were provided twice daily in a buffet style, allowing subjects complete freedom to choose their dishes and quantities. Training sessions started earlier, between 7:00 and 7:30 a.m., and the first meal was consumed after the morning training sessions. Similar to term A, aside from the scheduled meals, subjects freely consumed additional food and beverages.

BW and fat-free mass (FFM) were measured before each period using a weight meter (BC-315-WH; Tania Inc., Tokyo, Japan). FFM was estimated using eight skinfold measurements (triceps, subscapular, biceps, iliac crest, supraspinale, abdominal, thigh, and calf) according to the guidelines of the International Society for the Advancement of Kinanthropometry (ISAK) [15], performed by a level 1 ISAK-accredited anthropometrist. Self-reported height values were used. Subjects maintained detailed 24-h daily activity and training logs. All subjects followed individualized training plans prescribed by their coaches and spent their free time in their private rooms.

### 2.3. Energy and Macronutrient Intakes

Energy and macronutrient intakes were accurately assessed using the remote food photography method, which has proven reliable for measuring EI in free-living individuals [16]. The consumption of rice was quantified using a digital scale. A registered dietitian attended the training camp, documenting meal menus, ingredients, and cooking methods (e.g., boiling, baking, frying). Subjects also photographed and reported any snacks and beverages consumed outside of meals, including detailed information on quantities and brands. The dietitian reviewed and refined these records if necessary, and energy and macronutrient intakes were then calculated using specialized software (Excel Eiyo-kun ver. 9; Kenpaku-sha, Tokyo, Japan). The nutritional values of processed snack foods were determined from the nutrition fact labels on their packaging.

### 2.4. EEE and EA

Resting energy expenditure (REE) was estimated using the Cunningham equation [17], noted for its accuracy when applied to male endurance athletes [18]. Training activities were classified, and energy expenditures calculated, based on metabolic equivalents (METs) from the compendium of physical activities [19]. Energy expenditure for each activity was calculated as follows: Active energy expenditure = METs × weight (kg) × (min of activity/60). To avoid overestimation of EEE, the relative REE was subtracted from the total activity energy expenditure. The relative REE was calculated as follows [4,20]: Relative REE (kcal) = (REE (kcal)/24) × (min of activity/60). Therefore, EEE was calculated by: EEE (kcal) = activity energy expenditure (kcal) − relative REE (kcal). EA was then calculated as the difference between EI and the energy cost of exercise, relative to FFM, using the formula [21]: EA (kcal/kg FFM) = [EI (kcal) − EEE (kcal)]/FFM (kg).

### 2.5. IGC

IGC was continuously monitored using the FreeStyle Libre Flash glucose monitoring device (Abbott Diabetes Care, Alameda, CA, USA). Subjects wore the device from 11:00 a.m. the day before the experiment until 8:00 a.m. the following morning after the experimental period. The sensor was placed on the skin at the back of the upper arm and recorded average IGC values every 15 min [22]. To prevent data loss, an adhesive tarpaulin was applied over the sensor during activities such as swimming and bathing. Subjects scanned the sensors at least every 8 h, except during sleep. Data from 9:00 to 11:00 p.m. were excluded from the analyses because subjects typically slept > 8 h during this time. CGM provides glucose estimates comparable to self-monitoring blood glucose levels in normoglycemic individuals [23].

### 2.6. Statistical Analyses

Statistical analyses were conducted using SPSS software (ver. 28.0; IBM Corp., Armonk, NY, USA). Data are reported as mean ± standard deviation (SD). Daytime IGC was defined from 6:00 a.m. to 9:00 p.m., and nocturnal IGC from 11:00 p.m. to 6:00 a.m. The normality of the data was confirmed using the Shapiro-Wilk test. Correlations were assessed using Pearson’s correlation coefficient for normally distributed data and Spearman’s rank correlation coefficient for non-normally distributed data. A *p-*value < 0.05 was considered statistically significant.

## 3. Results

### 3.1. Energy and Macronutrients Intakes

Table 1 details the daily energy and macronutrients intake over 8 days (2 terms × 4 days) for subjects 1–3 and over 4 days for subjects 4 and 5. Overall, average CHO intake among the five subjects was 9.6 ± 1.7 g/kg BW/day, with notable inter-individual variation. CHO intake was >7 g/kg BW/day for all subjects except subject 4 on day A4 and subject 5 on day B1. Subject 3 consistently consumed > 10 g/kg BW/day of CHO during all 8 days, whereas subject 5 consumed < 10 g/kg BW/day during the 4-day period.

### 3.2. EEE

Table 2 presents the individual mean EEE and exercise duration. In addition to swimming, cycling, and running, strength training sessions were recorded for subject 1 on day B3 (0.37 h, 142 kcal) and for subject 4 on day A4 (1.5 h, 237 kcal). Among all exercise types, the most time was spent cycling.

### 3.3. EA

**Figure 1:**
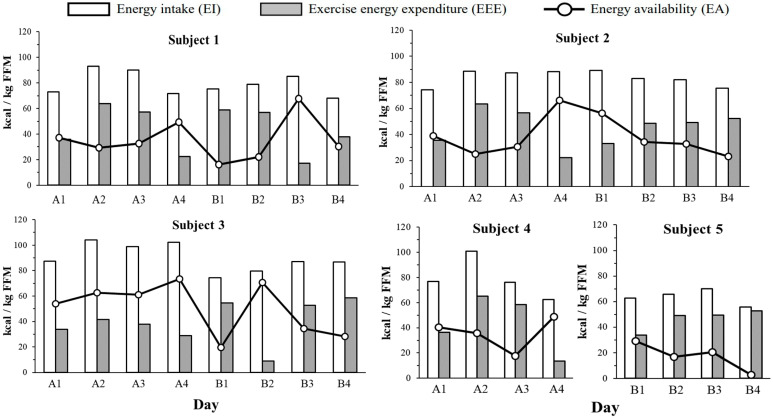
Individual daily energy intake (EI, white bars) exercise energy expenditure (EEE, gray bars) and energy availability (EA, circle markers) over 8 days (2 terms × 4 days) for subjects 1–3 and 4 days for subjects 4–5. All data were divided by FFM. EI; energy intake. EEE; Exercise energy expenditure. EA; Energy availability. FFM; Fat-free mass.

Figure 1 shows the EA, EI, and EEE, normalized to FFM, estimated prior to each period. EA was generally highest on days with the lowest EEE, except for subject 3, who showed an exceptionally high EA (70.5 kcal/kg FFM/day) on the day with the lowest EEE. For subject 5, EA remained < 30 kcal/kg FFM/day throughout the 4-day measurement period.

### 3.4. IGC Profiles

Table 3 summarizes the IGC data for each day. Daytime (6:00 a.m.–9:00 p.m.) IGC data include mean ± SD, coefficient of variation (CV), and maximum and minimum values, as well as the relative time (%) spent with ≥110 and 140 mg/dL. Nocturnal (11:00 p.m.–6:00 a.m.) IGC data include mean ± SD and minimum values, as well as the relative time (%) spent ≥ 100 mg/dL. During all measurement periods, IGC did not drop below 70 mg/dL for any subject. Additionally, the daily mean daytime IGC was ≥110 mg/dL on all days except for subject 1 on day A4.

### 3.5. Relationships of Nocturnal IGC with EA and EI

**Figure 2:**
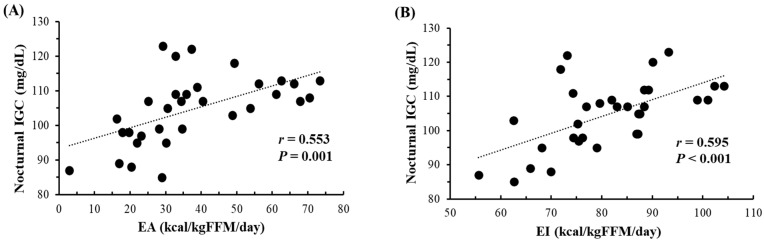
Relationship between nocturnal (11 p.m.–6 a.m.) IGC and both EA (**A**) and EI (**B**) (n = 32, over 8 days (2 terms × 4 days) for subjects 1–3 and 4 days for subjects 4–5). IGC; Interstitial fluid glucose concentration. EA; Energy availability. EI; Energy intake. FFM; Fat-free mass.

Figure 2 shows the correlations of daily mean nocturnal IGC with both daily EA and EI over 32 instances. During the measurement periods, subjects did not consume any food or beverages from 9:00 p.m. to 6:00 a.m. Significant correlations were observed between daily mean nocturnal IGC and daily EA (r = 0.553, *p* = 0.001), as well as EI (r = 0.595, *p* < 0.001). When the data (n = 32) were segregated into days with EA ≥ 30 kcal/kg FFM/day (n = 20) and <30 kcal/kg FFM/day (n = 12), days with EA < 30 kcal/kg FFM/day exhibited significantly lower nocturnal IGC compared to days with EA ≥ 30 kcal/kg FFM/day (97 ± 10 mg/dL vs. 109 ± 6 mg/dL, *p* < 0.001). However, no significant relationships were found between daily mean nocturnal IGC and daily CHO intake (g/kg BW) (r = 0.319, *p* = 0.075) or EEE (r = −0.278, *p* = 0.124).

### 3.6. Relationship between EEE and EI

**Figure 3:**
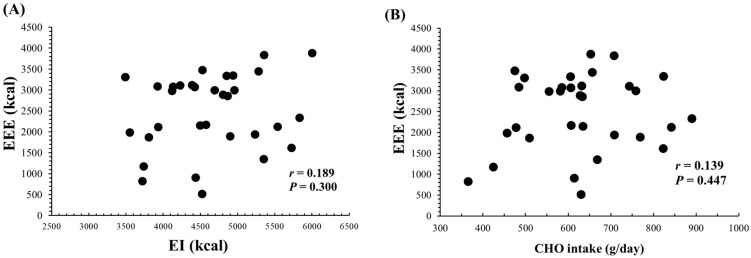
Relationship between daily EEE and both daily EI (**A**) or CHO intake (**B**) (n = 32, over 8 days (2 terms × 4 days) for subjects 1–3 and 4 days for subjects 4–5). EEE; Exercise energy expenditure. EI; Energy intake. CHO; Carbohydrate.

Figure 3 shows the relationships of daily EEE with both daily EI and CHO intake. There was no significant correlation between daily EEE and daily EI (r = 0.189, *p* = 0.300) or CHO intake (g/day) (r = 0.139, *p* = 0.447). Additionally, daily protein intake (g/day) (r = 0.094, *p* = 0.609) and fat intake (g/day) (r = 0.141, *p* = 0.441) also showed no significant correlations with daily EEE. 

## 4. Discussion

The present study examined how varying levels of EA affect 24-h changes in IGC during a training camp for elite Japanese male triathletes. A key finding was that daily mean nocturnal IGC significantly correlated with EA. Moreover, high daytime IGC levels were maintained without evidence of hypoglycemia, even on days with substantially reduced EA. This underscores the influence of EA on glucose regulation and highlights the resilience to varying dietary conditions of glucose levels in elite athletes.

### 4.1. Nocturnal IGC

The mean nocturnal IGC positively correlated with both daily EA and daily EI. However, we cannot strongly argue the relationship between nocturnal IGC and daily EA since we were only able to collect the data from five subjects. For subject 5, whose mean EA over 4 days was 17.3 ± 9.4 kcal/kg FFM/day, and daily mean nocturnal IGC was consistently lower compared to other subjects. An EA of 30 kcal/kg FFM/day is considered the threshold for LEA and is associated with an increased risk of relative energy deficiency in sport (REDs) symptoms in females [5,24]. In male athletes, the cut-off values for REDs-related symptoms are suggested to be lower than for females [13,14,25,26], although the precise values are still under discussion [27]. In exercising males, reductions of leptin and insulin were observed under EA of 15 kcal/kg FFM/day over 4 days [12]. We guess that the reason for the lower trend of nocturnal IGC for subject 5 compared to the other subjects is lowered EA.

Contrary to expectations, no subjects experienced hypoglycemia (<70 mg/dL) overnight, even when daily EA were extremely low. Previous research noted that elite endurance athletes under LEA (15.7 kcal/kg FFM/day) experienced more frequent nocturnal hypoglycemic episodes [11]. However, in sub-elite athletes, blood glucose levels rarely reached hypoglycemic levels unless calorie intake was extremely low [28]. Triathletes are typically advised to consume 8–10 g/kg BW/day of CHO to replenish glycogen stores during consecutive training days [29]. In the present study, on days when EA was <30 kcal/kg FFM/day, subjects consumed 6.9–13.3 g/kg BW/day of CHO. While some did not meet the recommended CHO intake levels, the CHO intake in the present study (6.9–13.3 g/kg BW/day) was generally sufficient to prevent nocturnal hypoglycemia.

The absence of hypoglycemia can also be attributed to the fact that the reduction in EA was not sustained. Heikura et al. [30] reported that no significant physiological disturbances occurred when EA fell to <15 kcal/kg FFM/day for a single day if the 8-day average EA was ~36 kcal/kg FFM/day in elite male cyclists. In the present study, although EA showed considerable day-to-day variability, the average EA for each period was ≥30 kcal/kg FFM/day for subjects 1–4; subject 5 was the exception, with an average EA of 17 kcal/kg FFM/day.

### 4.2. Daytime IGC

As anticipated, no instances of hypoglycemia were observed during daytime hours (6:00 a.m.–9:00 p.m.). Notably, the relative daytime IGC values were ≥110 mg/dL for >50% of the time, for all subjects except subject 1 on day B4. High-intensity exercise stimulates the secretion of catecholamines, which increases hepatic glycogen breakdown and leads to elevated blood glucose levels [31,32]. When hepatic glycogenolysis surpasses glucose uptake by muscles and liver, blood glucose concentrations rise during exercise [33]. Exercise-induced catecholamine levels are higher in trained athletes compared to untrained individuals [34], allowing athletes to maintain higher blood glucose levels even during prolonged exercise [35]. Furthermore, elite ultramarathon runners can avoid hypoglycemia during races, irrespective of EI [36]. Additionally, 24-h blood glucose concentrations in endurance athletes, who typically engage in longer training sessions and have a higher CHO intake, do not differ significantly from those in untrained males (7.4 vs. 7.3 mmol/L, respectively) [37]. Consistent with these findings, daytime IGC in the present study remained elevated, even though the average EEE was exceptionally high (4-day average range: 1989–2903 kcal/day).

Daily EEE did not show significant correlations with either daily EI or CHO intake. However, a significant negative correlation was observed between daily EEE and EA. Taylor et al. [38] noted a significant correlation between daily EEE and EI, though small increases in EI did not fully compensate for the increases in EEE in elite male road cyclists. Our results reinforce the observation that elite male triathletes often fail to increase EI proportionally to the rise in EEE. 

### 4.3. Limitations and Future Perspectives

The present study has several limitations. First, FFM was estimated using skinfold measurements, and EEE was calculated using METs. Although these methods are commonly used, they are not without flaws. A more accurate method for estimating EEE in field conditions might involve combining heart rate monitoring with laboratory-based indirect calorimetry [39]. Additionally, dual-energy X-ray absorptiometry (DXA) is the preferred method for more precise estimation of FFM [40].

Second, individual variations in blood glucose responses are significant [41,42]. Our data collection spanned only 4–8 days and involved only five subjects, which limits the generalizability of our findings. Monitoring IGC over longer periods and during different phases of athletic training (e.g., intensified training, tapering) could provide more detailed insights into the relationships among EA, training load, and nocturnal IGC.

Finally, glucose variability is influenced by numerous factors, including the timing and composition of nutrition, exercise intensity and duration, and physiological and psychological stresses. These interactions are complex and were not fully controlled in our study due to the naturalistic setting of an actual training camp. Additionally, patterns of LEA and low CHO availability can vary widely in the field, including subtle LEA over prolonged periods, repeated moderate LEA, and severe periodic LEA [38]. Future studies should consider conducting laboratory-based experiments under controlled environments to more accurately assess the effects of different LEA patterns on glucose dynamics.

## 5. Conclusions

Our findings provide a possibility that nocturnal IGC may be influenced by daily EA among elite male triathletes during a training camp. Despite varying EA levels, daytime IGC did not fall into the hypoglycemic range, underscoring the resilience to varying training conditions of glucose regulation in these athletes. However, because the present study was conducted as a case study with limited number of elite athletes, further determinations with large sample size are needed. Also, the data collection from female endurance athletes would be highly valuable.

## Figures and Tables

**Table 1:** Individual daily energy and macronutrients intake over 8 days (2 terms × 4 days) for subjects 1–3 and 4 days for subjects 4–5.

	Day	Energy	Carbohydrate			Protein			Fat	
		(kcal)	(g)	(g/kgBW)	for Energy	(g)	(g/kgBW)	for Energy	(g)	for Energy
Subject 1	A1	3808	509.5	8.8	53%	140.9	2.4	15%	134.0	32%
	A2	4855	605.6	10.5	50%	190.2	3.3	16%	185.8	34%
	A3	4693	581.5	10.1	50%	170.0	3.0	14%	187.4	36%
	A4	3740	425.0	7.4	46%	162.2	2.8	17%	154.6	37%
	B1	3925	484.7	8.5	49%	144.7	2.5	15%	156.4	36%
	B2	4124	555.6	9.7	54%	162.3	2.8	16%	139.1	30%
	B3	4439	614.9	10.8	56%	136.7	2.4	12%	159.2	32%
	B4	3554	456.7	8.0	51%	134.9	2.4	15%	131.9	34%
Subject 2	A1	4499	635.0	9.6	56%	177.4	2.7	16%	138.8	28%
	A2	5357	707.7	10.7	53%	229.3	3.5	17%	178.8	30%
	A3	5286	657.1	10.0	50%	220.0	3.3	17%	197.5	33%
	A4	5345	668.4	10.1	50%	221.4	3.4	17%	198.4	33%
	B1	5238	708.8	11.0	54%	201.5	3.1	15%	177.4	31%
	B2	4869	633.4	9.8	52%	178.3	2.8	15%	180.2	33%
	B3	4808	628.7	9.7	52%	159.9	2.5	13%	183.7	35%
	B4	4428	606.2	9.4	55%	152.0	2.4	14%	155.0	31%
Subject 3	A1	4902	769.3	12.3	63%	181.1	2.9	15%	122.3	22%
	A2	5834	890.4	14.2	61%	235.0	3.7	16%	148.0	23%
	A3	5539	842.4	13.4	61%	208.6	3.3	15%	148.2	24%
	A4	5727	823.4	13.1	58%	218.2	3.5	15%	173.4	27%
	B1	4229	744.0	11.9	70%	115.5	1.9	11%	87.9	19%
	B2	4526	630.6	10.1	56%	157.6	2.5	14%	152.6	30%
	B3	4959	759.1	12.2	61%	155.4	2.5	13%	144.5	26%
	B4	4914	824.2	13.2	67%	160.3	2.6	13%	108.4	20%
Subject 4	A1	4578	607.4	9.1	53%	209.0	3.1	18%	145.8	29%
	A2	6004	652.9	9.8	44%	244.8	3.7	16%	268.1	40%
	A3	4531	475.5	7.1	42%	209.3	3.1	18%	199.1	40%
	A4	3722	365.6	5.5	39%	167.5	2.5	18%	176.6	43%
Subject 5	B1	3935	478.2	6.9	49%	134.9	1.9	14%	164.7	37%
	B2	4133	585.2	8.4	57%	135.0	1.9	13%	139.1	30%
	B3	4389	632.1	9.1	58%	136.6	2.0	12%	146.0	30%
	B4	3491	498.1	7.2	57%	113.3	1.6	13%	116.2	30%

BW; Body weight.

**Table 2:** Individual mean EEE and duration of each exercise modality.

Subject		1		2		3		4	5
Days of Measurement		A1–A4	B1–B4	A1–A4	B1–B4	A1–A4	B1–B4	A1–A4	B1–B4
Mean EEE (kcal/day)	Total	2338 ± 866 (1169–3332)	2235 ± 881 (902–3080)	2692 ± 996 (1348–3835)	2686 ± 440 (1938–3068)	1989 ± 267 (1615–2332)	2489 ± 1148 (513–3342)	2584 ± 1198 (819–3874)	2903 ± 464 (2114–3307)
	Swimming	795 ± 192	547 ± 226	916 ± 218	840 ± 115	849 ± 212	659 ± 92	922 ± 226	905 ± 124
	Cycling	912 ± 651	1089 ± 788	1051 ± 748	1242 ± 692	536 ± 114	1169 ± 752	1015 ± 812	1340 ± 746
	Running	632 ± 130	564 ± 283	726 ± 151	605 ± 173	604 ± 80	661 ± 424	589 ± 362	658 ± 196
Mean DE (h/day)	Total	4.34 ± 1.12 (3.53–5.73)	4.31 ± 1.50 (1.85–5.85)	4.28 ± 1.19 (2.75–5.73)	4.04 ± 0.24 (3.63–4.20)	3.36 ± 0.51 (2.73–4.08)	3.56 ± 1.53 (0.93–4.68)	4.21 ± 1.28 (2.48–5.73)	3.99 ± 0.32 (3.43–4.20)
	Swimming	1.34 ± 0.34	1.11 ± 0.22	1.41 ± 0.29	1.31 ± 0.09	1.36 ± 0.34	1.04 ± 0.17	1.34 ± 0.34	1.31 ± 0.09
	Cycling	1.72 ± 0.87	1.98 ± 1.42	1.72 ± 0.87	1.83 ± 0.66	1.02 ± 0.15	1.48 ± 1.12	1.59 ± 1.05	1.83 ± 0.66
	Running	1.27 ± 0.25	1.07 ± 0.53	1.15 ± 0.22	0.91 ± 0.36	0.98 ± 0.09	0.83 ± 0.51	0.90 ± 0.56	0.86 ± 0.28

The values are mean ± SD. EEE; Exercise energy expenditure. DE; Duration of exercise.

**Table 3:** IGC data over 8 days (2 terms × 4 days) for subjects 1–3 and 4 days for subjects 4–5.

Subject 1	Day			A1	A2	A3	A4	B1	B2	B3	B4
	Daytime (6 a.m.–9 p.m.)	Mean ± SD (mg/dL)		147 ± 18	151 ± 22	145 ± 16	141 ± 16	120 ± 10	118 ± 13	112 ± 15	109 ± 11
		CV (%)		12.0	14.4	11.1	11.6	8.1	11.4	13.3	10.5
		Maximum (mg/dL)		197	197	178	178	151	160	153	140
		Minimum (mg/dL)		118	111	114	105	103	89	88	84
		≧110 mg/dL (%)		100	100	100	97	82	71	56	46
		≧140 mg/dL (%)		62	71	59	56	2	5	3	2
	Nocturnal time (11 p.m.–6 a.m.)	Mean ± SD (mg/dL)		122 ± 7	123 ± 8	120 ± 8	118 ± 9	102 ± 4	95 ± 2	107 ± 5	95 ± 5
		Minimum (mg/dL)		110	112	105	106	96	91	100	87
		≧100 mg/dL (%)		100	100	100	100	72	0	100	14
Subject 2	Day			A1	A2	A3	A4	B1	B2	B3	B4
	Daytime (6 a.m.–9 p.m.)	Mean ± SD (mg/dL)		138 ± 18	136 ± 17	134 ± 18	131 ± 17	136 ± 12	132 ± 18	127 ± 15	129 ± 16
		CV (%)		13.1	12.2	13.7	13.2	9.1	13.4	11.8	12.1
		Maximum (mg/dL)		189	175	199	188	161	176	164	179
		Minimum (mg/dL)		110	102	101	104	103	106	97	105
		≧110 mg/dL (%)		100	95	95	87	98	92	89	89
		≧140 mg/dL (%)		38	46	38	28	39	33	18	28
	Nocturnal time (11 p.m.–6 a.m.)	Mean ± SD (mg/dL)		111 ± 8	107 ± 6	105 ± 7	112 ± 7	112 ± 7	107 ± 7	109 ± 10	97 ± 3
		Minimum (mg/dL)		100	96	96	102	98	98	97	93
		≧100 mg/dL (%)		100	90	83	100	97	76	76	24
Subject 3	Day			A1	A2	A3	A4	B1	B2	B3	B4
	Daytime (6 a.m.–9 p.m.)	Mean ± SD (mg/dL)		142 ± 15	143 ± 19	143 ± 20	146 ± 20	128 ± 18	122 ± 16	121 ± 18	122 ± 16
		CV (%)		10.9	13.1	14.0	13.7	13.7	12.9	14.6	13.3
		Maximum (mg/dL)		181	187	189	184	158	161	159	166
		Minimum (mg/dL)		113	99	101	107	76	97	78	83
		≧110 mg/dL (%)		100	93	89	98	89	72	74	69
		≧140 mg/dL (%)		53	64	61	57	31	13	13	10
	Nocturnal time (11 p.m.–6 a.m.)	Mean ± SD (mg/dL)		105 ± 10	113 ± 9	109 ± 11	113 ± 10	98 ± 4	108 ± 11	99 ± 6	99 ± 5
		Minimum (mg/dL)		91	97	99	102	92	97	92	92
		≧100 mg/dL (%)		59	97	97	100	21	86	45	62
Subject 4	Day			A1	A2	A3	A4				
	Daytime (6 a.m.–9 p.m.)	Mean ± SD (mg/dL)		149 ± 24	141 ± 23	127 ± 22	127 ± 23				
		CV (%)		16.0	16.0	17.5	18.1				
		Maximum (mg/dL)		243	194	206	197				
		Minimum (mg/dL)		110	105	98	98				
		≧110 mg/dL (%)		100	98	85	77				
		≧140 mg/dL (%)		57	48	21	21				
	Nocturnal time (11 p.m.–6 a.m.)	Mean ± SD (mg/dL)		107 ± 6	109 ± 8	98 ± 6	103 ± 4				
		Minimum (mg/dL)		99	99	90	98				
		≧100 mg/dL (%)		90	97	24	76				
Subject 5	Day			B1	B2	B3	B4				
	Daytime (6 a.m.–9 p.m.)	Mean ± SD (mg/dL)		114 ± 20	121 ± 20	114 ± 16	116 ± 16				
		CV (%)		17.2	16.4	14.4					
		Maximum (mg/dL)		174	161	165	168				
		Minimum (mg/dL)		76	85	93	89				
		≧110 mg/dL (%)		51	71	53	71				
		≧140 mg/dL (%)		15	20	7	7				
	Nocturnal time (11 p.m.–6 a.m.)	Mean ± SD (mg/dL)		85 ± 9	89 ± 9	88 ± 6	87 ± 6				
		Minimum (mg/dL)		71	76	77	78				
		≧100 mg/dL (%)		10	14	7	3				

SD; Standard deviation. CV; Coefficient of variation.

## Data Availability

The data presented in this study are available on request from the corresponding author.
